# Memoir on the Morbific Elongation of the Tongue out of the Mouth

**Published:** 1801-10

**Authors:** Pierre Lassus


### *To the Editors of the Medical and Physical Journal.*

GENTLEMEN,

I Have translated the following Memoir from the first volume of the Memoirs of the National Institute of Sciences and Arts, in France; a scarce book in this country. This paper, among many others, appears interesting to me, both on account of the rarity of the subject therein treated, and for the practical utility it may afford. Its insertion in your useful Journal will oblige, Gentlemen, your's, &c.

*Plymouth, Aug.* 28, 1801. G. BELLAMY.

---

### *Memoir on the morbific Elongation of the Tongue out of the Mouth.* By PIERRE LASSUS.

THERE are some children of whom the point of the tongue tumefies, and is prolonged by little and little out of the mouth, extending extending even upon the chin. This vicious affection, which happily is rare, and the cause of which is not the same in all individuals, manifests itself sometimes immediately after birth, at others in the first years of infancy. In both cases this deformity, be it from birth or accidental, to which we may give the name of Prolapsus Linguæ, degenerates gradually when it is not remedied in its beginning, into an habitual disease, which increases with age; and that some of those who have been incommoded with it have preserved during life. As it does not appear that the medical profession has observed this disease with all the attention that it deserves, I have thought it might be useful to collect the few observations which have been made on this subject, to join them to those that I have had opportunity of making myself, to discuss the one and the other, so as to find the means of remedying this preternatural affection.* An author of the sixth century, Gaspar Peucer, is, as I believe, the first who has said he had seen children come into the world with the tongue out of the mouth, and hanging on the chin, like that of a calf recently slain. Such is the expression of Peucer, who considers this vicious conformation as a phænomenon, in some measure foreign to the art; as an incurable monstrosity.† The same case has been observed, with a little more exactness, by Zacchias;‡ this physician speaks of having seen, in 1628, at Rome, a new-born child, very strong and well formed, who had the tongue out of the mouth, the length at least of three fingers breadth; it was a little wider and a little thicker than it usually is at that age; when the child moved it, and drew it in, one could judge how much it exceeded the opening of the mouth.

Nevertheless, it sucked pretty well, provided the nurse's nipple was large and elongated; for it could not execute the same function with another nurse, whose nipple was rather short and thin. Arrived at the age of about fourteen months, it ate and drank pretty freely, although it had, night and day, a portion of the tongue out of the mouth; it began even to pronounce some words, when it died, without Zacchias having known the cause of its death.

The

---

* Sauvages has mentioned, in his Methodical Nosology, the involuntary exit of the tongue from the mouth, under the name of paraglosse exertoria, linguæ extrusio; but neither he, nor Gorter, nor Gaubius, whom he cites, has spoken of the disease which is the subject of this Memoir.

† Commentarius de Præcipuis Generibus Divinationum. Wittebergæ, 1580, in 8vo. p. 442. Teratoscopia.

‡ Quæstion. Medic. Legal. lib. vii. tit. 1. quæst. 9.

The following obfervation appears to prove, that this vicious conformation may exift even when the fœtus is ftill contained in the uterus. A woman, twenty-four years of age, was brought-to-bed, April 11, 1771, for the firft time, and being only in the eighth month of her pregnancy, of a dead fœtus, which was acephalous,* and whofe tongue, rather thick, projected about three lines out of the mouth.† The moft ufual cafes are thofe in which the prolongation of this organ manifefts itfelf immediately after birth, or a few years after; in fome children it is very confiderable at the moment of its appearance, whilft in others the progrefs of the difeafe is a little lefs rapid, and is only infenfibly advanced. An obfervation communicated to Bartholin, by Bogdan his difciple, proves this; who fays, he faw, at Driefen, in Brandebourg, a child which came into the world having the extremity of the tongue out of the mouth, of the fize of a filbert; this child receiving no help, its difeafe augmented fo, that it foon alarmed all thofe who beheld it. By little and little the tongue fwelled, and extended fo as to equal in volume the heart of a calf fix weeks old; which did not prevent this child, in the end, from fwallowing folid food, or from even fpeaking diftinctly, although the found of its voice was a little harfh.‡

It is certain that the difeafe increafes by fuffering the child to fuck, who cannot exercife that action but with difficulty; what proves this, is the neceffity of nourifhing it fometimes, by introducing liquid aliment very far into the mouth to be enabled to fwallow it. In proportion as the tongue extends and tumefies, it draws with it, by its weight, the hyoid bone, and the fuperior part of the larynx, which contributes to render deglutition ftill more difficult; the continual and very abundant effufion of faliva, which is no longer retained in the mouth, induces thirft and drynefs of the throat; the incifive and canine teeth of the lower jaw are thrown forward, and partly quit their alveoli; the tongue, rubbing againft thefe difplaced and worn teeth, excoriates and bleeds; the lower jaw, always hangs down, projects a little forward; the under lip reverfes, and projects; the fuperior edge of the lower jaw is hollowed by degrees in its middle, deprived at length of teeth by the preffure and motion of the tongue, which forms there a kind of furrow to lodge itfelf; in fine, this organ, at times more, at others lefs tumefied, hangs conftantly out of the mouth.

* The author muft mean the upper part of the head, the cranium, ftrictly fpeaking.

† Journal de Medicine, June, 1772. p. 498.

‡ Hiftor. Centur. III. Hiftor. 43, p. 84.

Such

Such are the fymptoms which characterife this difeafe when it is inveterate; at the fame time, it does not abfolutely prevent fpeech and deglutition; but the found of the voice is harfh, and deglutition is always more or lefs reftricted. *

The following obfervation will confirm all that has been faid on the progrefs that this difeafe is capable of making.

A woman of the town of Leyden had, in her infancy, a continued fever, which lafted fome weeks. At the time of her cure the tongue fwelled, and was elongated gradually out of the mouth, fo far as to hang on the chin, about the length of four inches; its width filled the whole interval of the angles of the lips.

The defire of hiding her deformity, or to make it lefs apparent, fuggefted to the girl, when fhe had obtained the age of fifteen years, the idea of enclofing her tongue in a little filver box, which did not prevent her from fpeaking, from forming founds, and executing the greater part of the functions attributed to this organ. At the age of fifty-four years fhe was examined (a little while fince) by the celebrated Sandifort, Profeffor of Anatomy at Leyden, who gives the following detail of the progrefs of this difeafe: The tranfverfe opening of the mouth is three inches, and is filled by the tongue, which hangs about the length of four inches and a half upon the chin; its furface is unequal, tubercular, and always covered with a thick faliva; the under lip is reverfed forwards, and lengthened three inches; its florid edge is not equal, but covered by two rounded eminences of near the thicknefs of the lip itfelf; the lower jaw is moveable, and conftantly hanging down by the weight of the tongue. She has only one tooth remaining, which is carious; one of the molars. In the roof of the palate are feen two roundifh tumors, feparated from each other by a fiffure. This difeafe is upwards of forty years ftanding, and caufes an almoft habitual and irkfome pain in the bottom of the throat, near the hyoides. During this long fpace of time, the patient has been often attacked with quinfey and difficulty of fwallowing. When fhe does not experience this inconveniene, fhe fwallows every kind of aliment, and fpeaks even pretty diftinctly. †

In cafes abfolutely like this, the cutting off the protuberant extremity of the tongue has been advifed, and even performed; the fear alone of a hæmorrhage, which has been thought difficult

* Journal de Medicine, Aout 1761, p. 156.

† Trioen, Obfervat. Medicinal. p. 142. Sandifort Obfervat. Anatom. Patholog. lib. 4. cap. 9.

cult to repreſs, has reſtrained the hand of ſome practitioners who were anxious to perform this uſeleſs operation. M. Sandifort, notwithſtanding, is of opinion, that it would be advantageous to the ſufferers, and honourable to the Art. We ſhall prove the contrary by and by; but let us firſt examine the fact upon which this learned Profeſſor eſtabliſhes his doctrine.

[ To be continued. ]

### *To the Editors of the Medical and Phyſical Journal.*

GENTLEMEN,

