# Errata

**Published:** 1802-10-01

**Authors:** 


					vol. vi. p. 350, 1. 40, ajter pel-formed, add{ in fpite of the art.

				

